# Future medical and non-medical costs and their impact on the cost-effectiveness of life-prolonging interventions: a comparison of five European countries

**DOI:** 10.1007/s10198-022-01501-6

**Published:** 2022-08-04

**Authors:** Hamraz Mokri, Ingelin Kvamme, Linda de Vries, Matthijs Versteegh, Pieter van Baal

**Affiliations:** 1 Erasmus School of Health Policy and Management (ESHPM), Erasmus University Rotterdam, Rotterdam, The Netherlands; 2 Institute for Medical Technology Assessment(iMTA), Erasmus University Rotterdam, Rotterdam, The Netherlands

**Keywords:** Economic evaluation, Future costs, Non-medical costs, Medical cost, Incremental cost-effectiveness ratio, Cost estimation

## Abstract

When healthcare interventions prolong life, people consume medical and non-medical goods during the years of life they gain. It has been argued that the costs for medical consumption should be included in cost-effectiveness analyses from both a healthcare and societal perspective, and the costs for non-medical consumption should additionally be included when a societal perspective is applied. Standardized estimates of these so-called future costs are available in only a few countries and the impact of inclusion of these costs is likely to differ between countries. In this paper we present and compare future costs for five European countries and estimate the impact of including these costs on the cost-effectiveness of life-prolonging interventions. As countries differ in the availability of data, we illustrate how both individual- and aggregate-level data sources can be used to construct standardized estimates of future costs. Results show a large variation in costs between countries. The medical costs for the Netherlands, Germany, and the United Kingdom are large compared to Spain and Greece. Non-medical costs are higher in Germany, Spain, and the United Kingdom than in Greece. The impact of including future costs on the ICER similarly varied between countries, ranging from €1000 to €35,000 per QALY gained. The variation between countries in impact on the ICER is largest when considering medical costs and indicate differences in both structure and level of healthcare financing in these countries. Case study analyses were performed in which we highlight the large impact of including future costs on ICER relative to willingness-to-pay thresholds.

**Supplementary Information:**

The online version contains supplementary material available at 10.1007/s10198-022-01501-6.

## Introduction

Cost-effectiveness analyses (CEA) are used to support decision-makers with decisions on the adoption of new healthcare technologies. The results of CEA are often presented as an incremental cost-effectiveness ratio (ICER), a ratio of incremental cost to incremental quality-adjusted life-years (QALY) gained by an intervention compared to an appropriate alternative [1–5]. Effective preventive and curative interventions can improve survival. Whilst increasing survival is a laudable result of any healthcare intervention, in the context of CEA, the fact that living longer results in more opportunity to consume healthcare and other goods and services (e.g., housing, electricity, water, and gas), must also be accounted for. The costs of this consumption in life-years gained (LYG) are referred to as future medical costs and non-medical consumption costs. Its inclusion may strongly impact upon CEA results, potentially even altering adoption decisions [4]. By definition, including these cost categories increases the ICER of life-prolonging interventions as each additional life-year carries additional costs.^1^ This has cast its inclusion in an unfavorable light, as if it would penalize successful healthcare interventions. Alternatively, interventions that improve quality of life become relatively more favorable when judged against the same cost-effectiveness threshold as life-extending interventions. On balance, it has been argued that consistent inclusion of future costs would support decisions that result in more health and welfare [1]. In this, future non-medical consumption is generally only relevant for CEAs conducted from a societal perspective.

Total future costs are the sum of future medical and future non-medical costs. Future medical costs are often divided into (i) medical costs related to the disease targeted by the intervention, related medical costs; and (ii) medical costs for all other medical consumption, unrelated medical costs [4]. For example, related medical costs of a heart failure intervention would include all healthcare costs associated with the treatment and subsequent costs of treating heart failure in later years. In this case, all healthcare costs unrelated to heart failure, such as the treatment of late onset diabetes, would be considered to be unrelated medical costs [1]. Future non-medical costs typically comprise non-medical consumption minus changes in production. Typically, only future related medical costs and productivity gains from living longer (for the societal perspective) are considered. Furthermore, only guidelines in the USA and the Netherlands explicitly recommend or require the inclusion of future unrelated medical costs and only guidance in the USA explicitly recommends including future non-medical costs [1]. Apart from theoretical discussions on the role of future costs in economic evaluations, an important reason for exclusion is a practical one: the scarcity of readily available estimates and tools that facilitate inclusion. To overcome this practical barrier, we present and compare estimates of future costs for five European countries: the Netherlands, Germany, the United Kingdom (UK), Spain, and Greece. We then demonstrate the impact that inclusion of these costs has on the cost-effectiveness of life-prolonging interventions.

The estimation of future unrelated medical costs is not without difficulties. As early as 1997, David Meltzer (1997) suggested to utilize healthcare expenditure (HCE) data by age which contain medical spending on all types of diseases [4]. Cost profiles can further be linked to survival curves to estimate the incremental cost impact of gained life-year in an intervention [1]. Furthermore, de Vries et al. suggest correcting the medical cost profiles for cost of related diseases to avoid double counting when deriving future unrelated medical costs [1, 6–8]. Future unrelated medical costs estimations can be further refined by considering the effect of time-to-death (TTD)—the observation that individuals consume most healthcare in the last year of life [9]. Building on this methodology, researchers have estimated country-specific standardized estimates of unrelated medical costs per additional year of life gained from an intervention. In the Netherlands, Van Baal and colleagues produced the Practical Application to Include Disease costs (PAID) tool based on the Dutch cost-of-illness (COI) study of 2005 and provide age- and sex-specific HCE attributable to disease categories [9], work that was recently updated [10]. Perry-Duxbury and colleagues developed a similar tool using estimates based on a combination of individual-level NHS inpatient data and aggregate NHS spending on outpatients in 2011/12 [11, 12]. Country-specific tools or comparable guidance for future medical costs for other countries could be developed as well as necessary estimates of age-, sex-, and disease-specific spending [9, 11, 13, 14]. Like future medical costs, non-medical costs can be derived from non-medical costs by age data. Importantly, as non-medical costs may be based on household consumption estimations, they require adjustments to reflect the composition of household. In his 1997 paper, Meltzer presented estimates of the magnitude of future medical and non-medical costs by age, and demonstrated the effect of inclusion in cost-effectiveness analyses. The empirical results in Meltzer’s paper suggests future costs could substantially alter cost-effectiveness of common medical interventions, especially if the intervention increases survival more than quality of life [4]. Further, non-medical costs by age have been estimated for the Netherlands by Kellerborg et al. (2021) [15] and added to the PAID tool, using individual-level data from a cross-sectional Dutch Household Consumption survey from 2004. Utilizing these data, they estimated non-medical costs for an average household by age. Furthermore, they accounted for economies of scale in consumption within households [10].

Previously, studies demonstrated the inclusion of future costs in economic evaluations in several countries, such as England and Wales, the Netherlands, and the United States (U.S.) [13, 14, 16–20]. These studies focus on the importance of including future costs in economic evaluation. In addition, they demonstrate that adjusting unrelated medical costs for time-to-death can have a significant effect on the cost-effectiveness ratio. Furthermore, the impact of including these costs in economic evaluations has been studied in disease areas such as diabetes mellitus, chronic heart failure and chronic kidney disease [21–27]. However, to our knowledge, there are no studies with empirical estimates of future costs investigating cross-country comparisons.

In this paper, we estimate and compare future costs of five European countries: the Netherlands, Germany, the UK, Spain, and Greece. For the Netherlands, both unrelated medical costs^2^ and non-medical cost^3^ have already been produced. For the UK, unrelated medical costs were already produced.^4^ For the estimation of future costs in the remaining countries, we use the methodology of van Baal et al. [9] and Kellerborg et al. [10] as a starting point but adapted the cost derivation approach in some cases due to limited data availability, see Table 1. Furthermore, we estimate the impact of including these costs on the cost-effectiveness of life-prolonging interventions in a general sense, and in a specific heart failure case study.

**Table 1:** Cost data input overview of sources by country and cost type

	The Netherlands	United Kingdom	Germany	Spain	Greece
UMC Data sources	Year: 2017 Type: Dutch cost-of-illness (COI) aggregate healthcare cost data stratified by 5-year age profiles, sex, healthcare provider, and disease categories [9, 10] Components: Inpatient and outpatient care, long-term care, retail sale and providers of medical goods, and other healthcare providers	Year: 2011 Type: Aggregate Hospital Episode Statistics and general practitioner visits for each age up to 84 (average estimate for age 85+) and sex [11, 12] Components: Inpatient and outpatient care, and GP and pharmaceutical spending	Year: 2015 Type: German COI aggregate healthcare cost data by 5- and 15-year profiles, sex, and disease categories [41, 42] Components: Inpatient and outpatient care, pharmaceutical-, medical remedies-, and aids spending [43]	Year: 2008 Type: Aggregate healthcare cost (Public Expenditure on Health (EGSP)) data by 5-year age profiles (average estimate for age 80+) and sex [44]*.* Public expenditure of EGSP and SHA [40]. Heart failure related costs [45] Components: Inpatient, specialized outpatient, prescription drugs, transport, and other healthcare categories	Year: 2014 Type: Aggregate hospital expenditures, and hospital admission frequency data as hospital consumption by 10-year profiles (an average estimate for age 75+) and sex [46–48]*.* Heart failure related costs [49] Components: Inpatient care
UMC Method	Van Baal et al. (2011) and Kellerborg et al*.* (2020) interpolated per capita healthcare spending by age using smoothing functions. Costs by age were decomposed into decedent and survivor costs using Dutch decedent to survivor cost ratios stratified by disease and healthcare provider. Details on methods and assumptions listed elsewhere [9, 10]	Perry-Duxbury et al. (2020) interpolated per capita healthcare spending by age using smoothing functions. Costs by age were decomposed into decedent and survivor costs using UK decedent to survivor cost ratios. Details on methods and assumptions listed elsewhere [11]	We interpolated per capita healthcare spending by age using smoothing functions. Costs by age were decomposed into decedent and survivor costs using Dutch decedent to survivor cost ratios	We adjusted EGSP estimates to SHA and interpolated per capita healthcare spending by age using smoothing functions. Cost by age were decomposed into decedent and survivor costs using Dutch cost decedent to survivor cost ratios	We estimated hospital expenditures per capita by age and sex, where hospital stays and costs per hospital day were based on hospital and admission data. Costs per capita were interpolated by age using smoothing functions and decomposed into decedent and survivor costs using Dutch decedent to survivor cost ratios
NMC Data sources	Year: 2004 Type: Household-level data on expenditures from the cross-sectional Dutch Household Consumption survey Components: All household purchases such as rent, food, clothing, and transport	Year: 2018–2019 Type: Household-level data on expenditures from the Living Costs and Food Survey (LCF) [50] Components: All household purchases such as rent, food, clothing, and transport	Year: 2019 Type: Aggregate-level data on average household consumption in 10-year age profiles of the main breadwinner. From a sample of income and household expenses for private consumption [51] Components: All household purchases such as rent, food, clothing, and transport	Year: 2010 Type: Aggregate-level data on average household consumption by 10- and 15- year age profiles of the main breadwinner. From the household Budget Survey Survey (HBS) [52] Components: All household purchases such as rent, food, clothing, and transport	Year: 2019 Type: Aggregate-level data on monthly average purchases and receipts in households classified by 10-year age profiles of the main breadwinner. From the household Budget (HBS) [53] Components: All household purchases such as rent, food, clothing, and transport
NMC Method	Kellerborg et al*.* (2020) estimated average NMC per capita by age using two generalized additive models with penalized B-splines on age. The first model estimated annual consumption per household equivalent. The second model estimated the probability of a household having more than 1 adult. These regression models were combined to estimate marginal consumption due to a death prevented in an average household. Details on methods and assumptions are listed elsewhere [10]	We estimated average NMC by age using two generalized additive models with penalized B-splines on age. The first model estimated annual consumption per household equivalent. The second model estimated the probability of a household having more than 1 adult. These regression models were combined to estimate marginal consumption due to a death prevented in an average household. Methods and assumptions are identical to those listed elsewhere [10]	We estimated average NMC per household equivalent by age category of the breadwinner. Costs are adjusted for average household size and economies of scale within households for the first and second adult. Data on the probability of having more than one adult per household was adopted from UK data. We used a smoothing function to interpolate the age profiles (20–80)	We estimated average NMC per household equivalent by age category of the breadwinner. Costs are adjusted for average household size and economies of scale within households for the first and second adult. Data on the probability of having more than one adult per household was adopted from UK data. We used a smoothing function to interpolate the age profiles (continuous 20–64, constant 65–80)	We estimated average NMC per household equivalent by age category of the breadwinner. Costs are adjusted for average household size and economies of scale within households for the first and second adult. Data on the probability of having more than one adult per household was adopted from UK data. We used a smoothing function to interpolate the age profiles (continuous 20–69, constant 70–80)

## Methodology

### Conceptual model

In the theoretical model of Meltzer, the incremental costs in an ICER are seen as the difference in consumption minus production between two interventions.^5^ The costs of consumption can be further categorized into related and unrelated medical costs (RMC, UMC), as well as non-medical costs (NMC) as presented in Eq. 1, where the future costs are only affected by changes in survival through a change in life-years (LY):

1 $$\mathrm{ICER}=\frac{\Delta \left[\mathrm{LY}\times \left(\mathrm{RMC}+\mathrm{PC}\right)\right]}{\Delta \mathrm{ QALY}}+\frac{\Delta \mathrm{ LY}\times (\mathrm{UMC}+\mathrm{NMC})}{\Delta \mathrm{ QALY}}.$$

Related medical costs and productivity costs (PC) as presented in the first part of the equation are typically already included in CEA under the societal perspective [4, 10, 18]. Hence, in this paper, we focus on the second part of the equation, which captures unrelated medical and non-medical costs. In this study, we use MC estimations as a substitute for UMC. This implies some double counting of related costs, although the overestimation is expected to be small [28]. Comparing the sizes of related and unrelated medical costs of heart failure in the PAID3.0 tool, shows the related costs can be mere fractions of the unrelated costs. For example, in the case of a 65-year-old male heart failure patient, the ratio of related to unrelated medical costs is about 0.002 [10]. We present the results of unrelated medical and non-medical costs of the equations separately. These ‘partial ICERs’ can be interpreted as the incremental unrelated medical and non-medical costs per QALY gained resulting from a hypothetical intervention in which a death at a certain age is prevented at zero intervention costs. Previous empirical research has shown that such ‘partial ICERs give a good indication of the impact of including future costs ICERs for non-hypothetical interventions [4, 10].

The methodology applied in this study was developed by Van Baal et al. [9] and builds on the theoretical foundations of Meltzer (1997). Following their approach, the estimation of future costs was modeled as future UMC and NMC (LY × [UMC + NMC]) for an individual as the lifetime costs of the individual of age a dying at age n as seen in Eq. 2.Where possible, unrelated medical costs can be derived from medical costs by extracting disease related costs. This is illustrated in the Eq. 2 as the medical costs of all unrelated diseases *i.* It has been demonstrated that time-to-death is a stronger predictor of HCE than age, coined as the red herring theory [29, 30]. This can be accounted for by stratifying unrelated medical costs into costs in the last year of life (decedent costs denoted *DC*) and costs in other years (survivor costs denoted *SC*) in Eq. 2. As such the equation illustrates the survival and decedent costs of all unrelated diseases $S{C}_{i}$ and $D{C}_{i}$, and non-medical costs *NMC* for the life-years remaining:

2 $$\mathrm{LY}\times \left[\mathrm{UMC}+\mathrm{NMC}\right]=\sum_{a}^{n-1}\sum_{i}{\mathrm{SC}}_{i}\left(a\right)+\sum_{i}{\mathrm{DC}}_{i}\left(n\right)+\sum_{a}^{n}\mathrm{NMC}\left(a\right).$$

Although it could be argued that non-medical costs can be different for different health states, the evidence for this is, as yet, inconclusive [10]; we, therefore, assume that non-medical costs are unaffected by disease. Non-medical costs are calculated as non-medical consumption per household equivalent; this calculation is achieved by adjusting household consumption for economies of scale within households in terms of consumption. This means that marginal consumption by each additional household member is reduced as the household size grows, reducing the costs of consumption per person when the household size is larger [31]. Applying the economies of scale concept to future non-medical costs indicates that preventing a death in a single-person household will result in more future non-medical consumption than preventing a death in a multi-person household [32]. Equation 3 shows the model that is used to estimate average annual non-medical consumption by age, when a death in an average household is prevented:

3 $$\mathrm{nmc}\left(a\right)=\left[hh \,equiv\left(a\right)\times h\left(a\right)\times w\right]+\left[hh \,equiv\left(a\right)\times \left(1-h\left(a\right)\right)\right].$$

where *h* is the probability of a household having more than one adult *and hh equiv* shows the annual non-medical consumption per household equivalent. In this equation, *w* is the consumption share of a household member: 0.5 for an adult and 0.3 for a child. The probability of households having more than one adult is estimated from the UK data and replicated for Germany, Greece, and Spain, due to the unavailability of the necessary data in these countries. While unrelated medical costs can be decomposed for sex, non-medical costs cannot, and hence are only presented by age.

We calculate the impact of future costs on the ICER of life-extending treatments by dividing the costs over the life-years gained, as seen in Eq. 4:

4 $$\frac{\Delta \mathrm{ LY}\times (\mathrm{UMC}+\mathrm{NMC})}{\Delta \mathrm{ QALY}}= \frac{{\sum }_{a}L\left(\mathrm{age}\right) \times \mathrm{ umc}\left(\mathrm{age}\right)}{{\sum }_{a}L\left(\mathrm{age}\right)\times \mathrm{QoL}\left(\mathrm{age}\right)}+ \frac{{\sum }_{a}L\left(\mathrm{age}\right) \times \mathrm{ nmc}\left(\mathrm{age}\right)}{{\sum }_{a}L\left(\mathrm{age}\right)\times \mathrm{QoL}\left(\mathrm{age}\right)},$$

where $\mathrm{L}\left(\mathrm{age}=a\right)$ are the life-years gained at age $a$. Similarly, $\mathrm{QoL}(\mathrm{age}=a)$ is the average quality of life at age $a$*.* In this study, we derive $\mathrm{L}(\mathrm{age}=a)$ from mortality rates in The Human Mortality Database [33]. The country-specific^6^ EQ-5D-3L Health-Related Quality of Life estimates by age were sourced from Heijink et al*.* 2011 [34]. As countries differ in their guidance on discount rates analyses were performed for three different sets of discount rates. The sets of discounting rates were based on the guidance for the countries of this study. Guidance within the UK proposes a 3.5% discount rate [11], while German and Spanish guidelines suggest 3% discounting for both cost and effect. Dutch guideline, on the other hand, suggests different discounting levels for cost and effects, namely 4 and 1.5%, respectively [35, 36]. No specification for discount rates was found for Greece. The results of the future costs impact analyses are presented in Table 2.

**Table 2:** Estimated medical, non-medical and combined impact on ICERs at different cost and effects discounting rates^a^

	The Netherlands (€)	United Kingdom (€)	Germany (€)	Spain (€)	Greece (€)
Unrelated medical costs					
At age 25					
3.5%	5200	3200	3600	2300	500
4% (costs) and 1.5% (effects)	3000	1900	2100	1400	300
3%	5500	3300	3800	2400	500
At age 50					
3.5%	8800	5500	6700	3200	600
4% (costs) and 1.5% (effects)	6000	3900	4600	2200	400
3%	9100	5600	6900	3200	600
At age 65					
3.5%	14,200	8100	10,400	4000	800
4% (costs) and 1.5% (effects)	11,000	6400	8200	3100	700
3%	14,600	8100	10,600	4000	900
At age 75					
3.5%	22,200	10,100	14,500	4900	1000
4% (costs) and 1.5% (effects)	18,800	8600	12,300	4100	800
3%	22,500	10,100	14,600	4900	1000
Non-medical costs					
At age 25					
3.5%	10,900	14,000	13,700	11,600	8400
4% (costs) and 1.5% (effects)	6800	8800	8600	7200	5300
3%	11,000	14,100	13,900	11,700	8400
At age 50					
3.5%	12,200	15,800	16,600	13,000	9000
4% (costs) and 1.5% (effects)	9200	11,900	12,500	9800	6800
3%	12,200	15,800	16,600	13,000	9000
At age 65					
3.5%	12,400	16,100	17,400	14,200	9200
4% (costs) and 1.5% (effects)	10,700	13,900	14,900	12,200	7900
3%	12,400	16,100	17,400	14,200	9200
At age 75					
3.5%	12,600	15,200	17,800	15,700	9600
4% (costs) and 1.5% (effects)	11,900	14,300	16,900	14,800	9100
3%	12,600	15,100	17,800	15,700	9600
Combined UMC and NMC					
At age 25					
3.5%	16,200	17,100	17,300	13,900	8800
4% (costs) and 1.5% (effects)	9900	10,700	10,700	8600	5600
3%	16,500	17,400	17,700	14,000	8900
At age 50					
3.5%	21,000	21,300	23,300	16,200	9600
4% (costs) and 1.5% (effects)	15,600	15,800	17,100	12,000	7200
3%	21,300	21,400	23,500	16,300	9600
At age 65					
3.5%	26,700	24,200	27,900	18,200	10,000
4% (costs) and 1.5% (effects)	20,200	20,200	23,100	15,300	8500
3%	27,000	24,300	28,000	18,200	10,000
At age 75					
3.5%	34,800	25,200	32,400	20,500	10,600
4% (costs) and 1.5% (effects)	23,500	22,900	29,200	19,000	9900
3%	35,100	25,300	32,500	20,600	10,600

All results are rounded to the nearest hundredth

^a^Based on country-specific guidelines; UK discount rate of 3.5% for both cost and effects; NL discount rate of 4% for costs and 1.5% for effects; Germany and Spain discount at 3% for both costs and effects [35, 36]

We demonstrate the impact of including future costs in the ICER in a case study. Unrelated medical and non-medical costs results are included in a cost-effectiveness model for the Sacubitril/Valsartan treatment for Heart Failure Patients in the Netherlands by van der Pol et al. 2021 [37]. Van der Pol and colleagues performed a headroom analysis which included future costs; however, the outcome of this analysis was a maximum daily drug cost for a specific willingness-to-pay (WTP) threshold. The maximum daily drug costs cannot be used to demonstrate the incremental impact of including unrelated medical and non-medical costs on the ICER. We therefore use Eq. 4 to enable the inclusion of future costs in the CEA model, which allows the incremental impact on the ICER to be showcased. We assumed the increased survival and QoL in the Sacubitril/Valsartan model as a proxy for the effectiveness of the intervention across countries, allowing us to estimate the future cost impact in other countries as well.

### Estimating cost profiles

Average healthcare expenditures per capita and household expenditures decomposed by relevant demographic characteristics like age and sex were preferred. Where such data were unavailable, the approach for deriving costs was adjusted depending on the type of the data found; for example, Greek medical costs by age were derived from aggregate expenditure and hospital admission data. Specific descriptions of the input data found, and methods used to derive costs in each country are presented in Table 1. The table includes the estimates previously produced by van Baal, Perry-Duxbury, and Kellerborg and colleagues for the Netherlands (PAID 3.0) and UK (PAIDUK 1.0). For the sake of comparison, PAID 3.0, PAIDUK 1.0 and other countries’ estimates were adjusted to 2020 price levels, and in the case of estimates sourced for PAIDUK 1.0, British Pound estimates were converted to Euros [38].

Medical costs based on aggregate healthcare data or hospital expenditure data combined with admission frequency data^7^ were used to estimate average healthcare costs per capita by age for both males and females. The comparability between countries is complicated by different cost components within healthcare costs. For example, Greek healthcare costs only include inpatient costs; for the other countries, medical expenditures include more cost components, such as costs of general practitioners. Healthcare expenditure data in Greece and Germany were based on System of Health Accounts (SHA), while the Spanish data were based on Public Healthcare Expenditure Statistics (EGSP) [39]. The Spanish costs were therefore inflated to the SHA levels in the country [40]. We were not able to adjust the unrelated medical costs of the Netherlands nor the UK, which were based on National Institute for Public Health and the Environment (RIVM) and Hospital Episode Statistics (HES), respectively. We exclude healthcare costs insurance premiums from non-medical costs to avoid double counting. Non-medical costs for Germany, Spain and Greece were based on aggregate-level data on average household consumption by age profiles of the main breadwinner, while non-medical costs for the UK were based on individual-level data on household expenditures. For Germany, Greece, and Spain we did not have data on household composition on an individual level, so averages by age were used to convert household consumption into consumption per household equivalent. Data on the probability of having more than one adult per household were assumed to be equal to those from the UK for these countries.

**Fig. 1:**
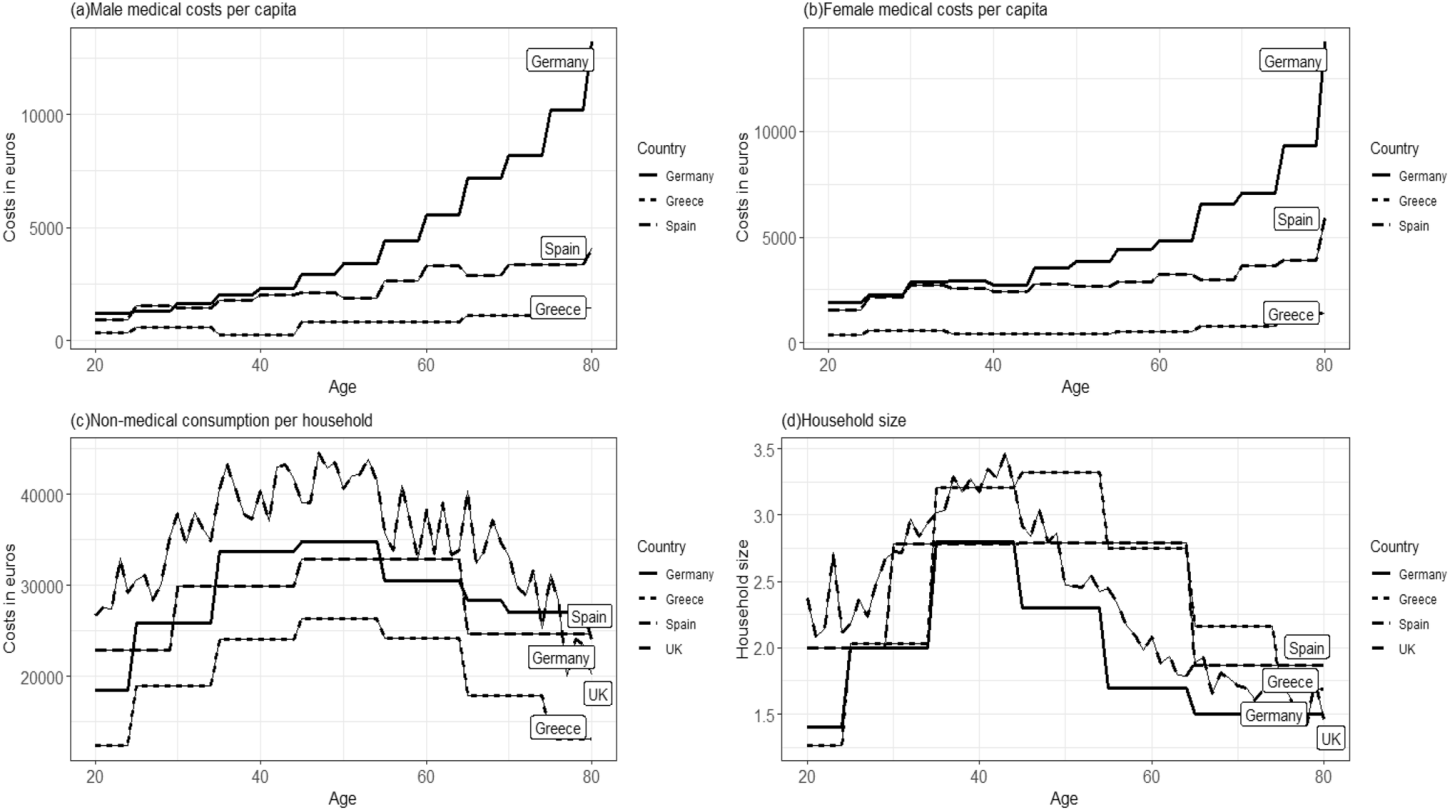
Average UMC per capita by age and gender (**a** and **b**), and average NMC (**c**) and average household size (**d**) by age of the main breadwinner. All costs are adjusted to €2020 price levels. *DE* Germany, *GB* United Kingdom, *ES* Spain, *GR* Greece

The cost inputs for medical and non-medical expenditures are presented in Fig. 1. The upper row of this figure displays medical expenditures by age for males and females separately, while the bottom row displays non-medical input data (not adjusted for per household and household size). It shows that female unrelated medical costs are somewhat higher than those for male for several age groups, specifically in mid-life and at older ages, yet this condition does not hold for all age groups; nor is it the case for Greece. In general, German and UK non-medical cost inputs appear remarkably larger than those for Spain and Greece. The difference in cost inputs between countries can partially be explained by the variation of cost components in the inputs, as is shown in Table 1. Graph (d) shows that the average household size is largest when the main breadwinner is of age 40–50 years, with slight differences between countries.

Cost inputs were interpolated using cubic splines, assuming average midpoints of the age groups for interpolating the costs. Due to limited cost data in some countries, we limit the costs of medical and non-medical consumption data to the working ages of 20–80. Medical costs can be corrected for country-specific related medical costs. As we use medical costs as a substitute for unrelated medical costs, the results are unadjusted as disease-specific costs. The unrelated medical costs used in the case study are derived using country-specific heart failure related costs. Heart failure related mortality and QoL data, and effectiveness of the Sacubitril/Valsartan treatment were available in the CEA model [37]. In the results section, all reported numbers are rounded to hundredths.

## Results

**Fig. 2:**
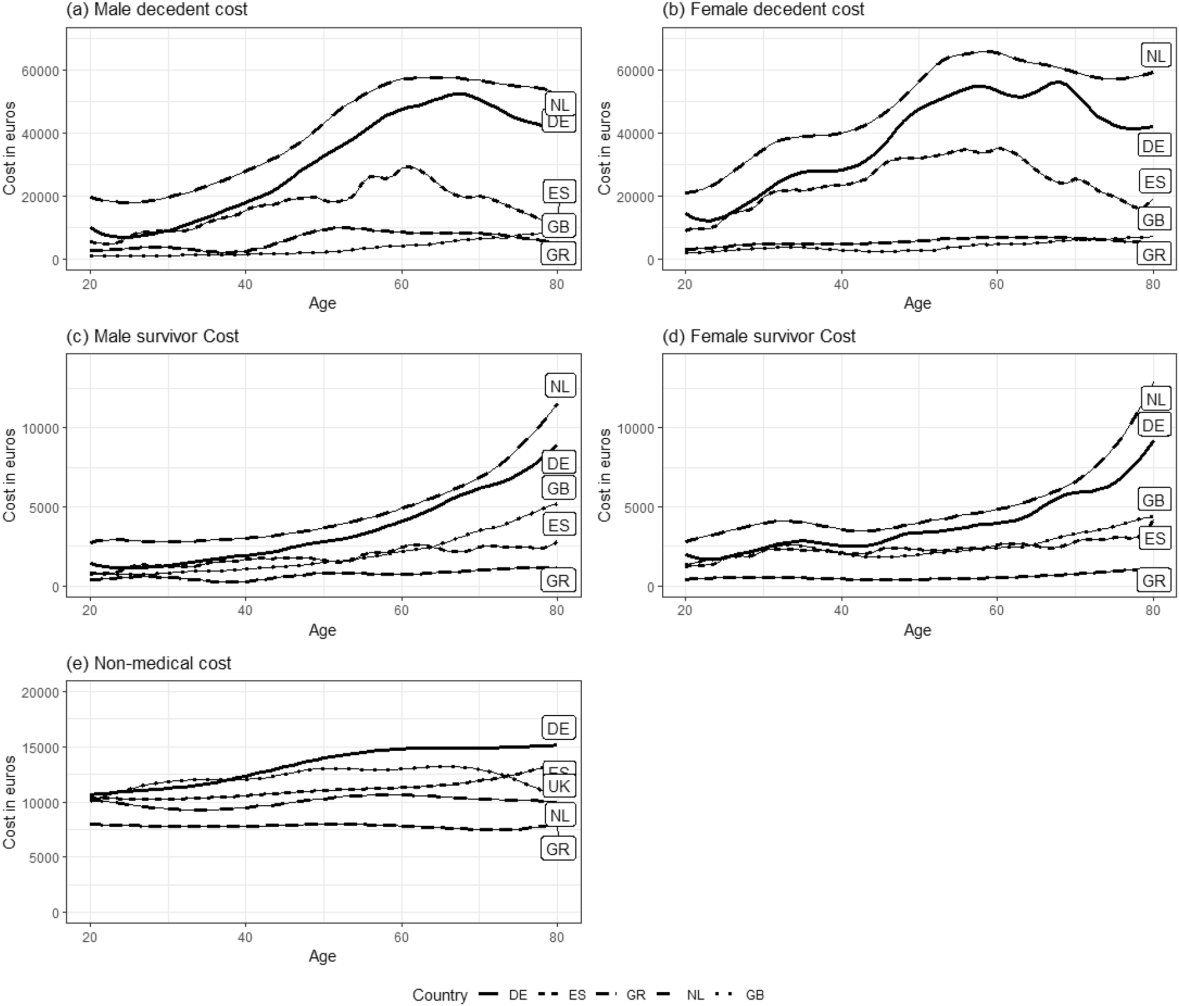
Estimates of annual medical per capita and non-medical per household costs in 2020, where UMC is decomposed into decedent and survivor costs by sex. NMC are per household equivalent by age of the main breadwinner, where costs for the UK and NL are per capita and by age of main breadwinner. *NL* The Netherlands, *DE* Germany, *GB* United Kingdom, *ES* Spain, *GR* Greece

Figure 2 shows the absolute unrelated medical and non-medical costs for all five countries as costs by age profile. The first two rows show medical decedent and survivor costs for male (left) and female (right), respectively. Note that the Y-axis scale varies between the graphs for decedent and survivor costs. As expected, these graphs show that age can be linked to the magnitude of both types of costs, albeit in somewhat different patterns across both the cost-types and countries. Both survivor and decedent costs generally increase with age, although decedent costs peak around age 60 with slight fall in costs past this age; the exception to this is the female German decedent costs, which have another peak around age 70. Therefore, despite adjusting for TTD, survival costs still rise significantly at higher ages for the Netherlands and Germany, and to a lesser extent for the UK, while remaining relatively stable over age in Spain and Greece. Decedent costs are relatively small for the UK, when compared to survivor costs. Notably, the ratios of costs in the last year of life and years survived for the UK are remarkably smaller than the Dutch ratios applied to the German, Spanish, and Greek unrelated medical costs. The decedent to survivor cost pattern is influenced by the cost ratio, but also the size of the cost inputs. Hence, the large input values of German unrelated medical costs are amplified more in the results relative to the low Greek and Spanish unrelated medical costs input values. This is especially visible for older age groups.

The third row in Fig. 2 shows average annual non-medical costs per household equivalent and by the age of the main breadwinner for each country. The average annual non-medical costs per household equivalent in Germany, Spain and Greece are around €13,300, €11,100, and €7800, respectively. For the Netherlands and the UK, the average non-medical costs by age are around €10,000 and €12,200, respectively. The shapes of the non-medical costs by age graphs differ slightly per country. For the UK, the costs increase up to age 70 and decrease thereafter. For the Netherlands the costs increase up to age 60 and decease thereafter. Whereas, for Spain and Germany the cost keeps increasing. Non-medical costs for Greece remain almost constant for all ages. The differences in non-medical costs per country might be because the cost for housing, clothing, transport, etc. are higher in some countries than others. The differences in estimated non-medical costs between countries can be explained by the inter-country variation in the average household size, purchasing habits and incomes. For example, the disposable income per capita in 2019 in Germany and the Netherlands was around €44,000 PPP and €39,000 PPP. Whereas, in Spain and Greece, the disposable income per capita per capita was €30,000 PPP and €23,000 PPP, respectively [54]. However, the price levels for consumer goods and services for the Netherlands and Germany are 20 and 8% above the EU average, leading to higher expenses per household [55].

**Fig. 3:**
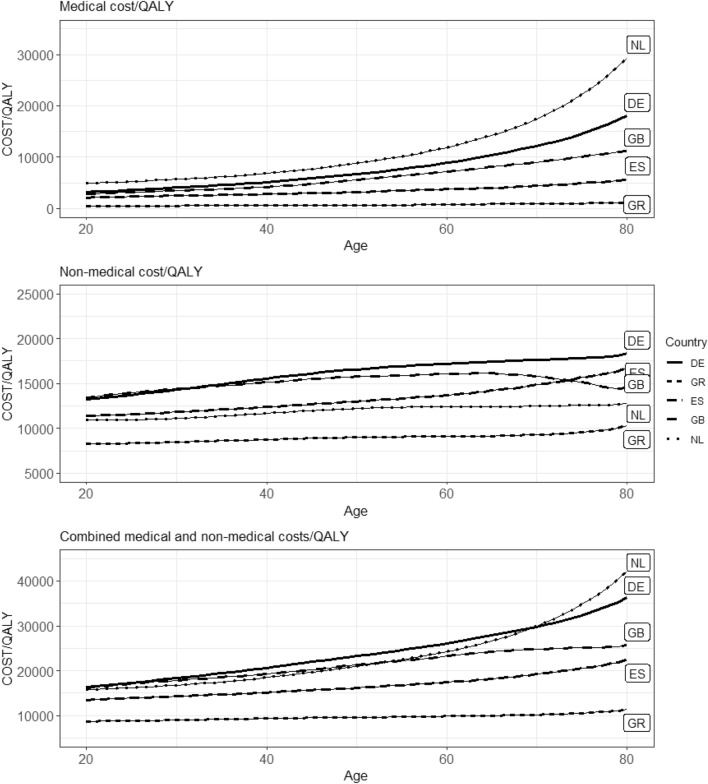
Impact of future UMC, NMC, and both on the ICER of life-saving interventions when assuming UK discount rates of 3.5% for both costs and effects. *NL* The Netherlands, *DE* Germany, *GB* United Kingdom, *ES* Spain, *GR* Greece

The impact of adding future costs to the ICER of life-saving interventions (i.e., avoiding death at that age) is shown in Fig. 3. The ICER analyses were based on Eq. 4 and derived using the absolute future costs in Fig. 2. We used an average of the unrelated medical costs for males and females unadjusted for TTD. The ICER analyses in Fig. 3 were run using the UK-based discount rates (3.5%) for both costs and effects. Analyses using the other discount rates are presented in Table 2. The estimates in Fig. 3 (and Table 2) can be interpreted as the ‘partial ICER’ (as described in the conceptual model section) when implementing the future costs by age in a hypothetical life-saving intervention.

The upper graph of Fig. 3 shows the impact of including future unrelated medical costs, demonstrating a similar pattern of gradually increasing impact with age, as well as a similar ordering of countries, when compared to medical costs in Fig. 2. The impact at lower ages is relatively similar for the different countries. As individuals approach older ages the differences in the impact of unrelated medical costs between countries becomes relatively large, ranging from about €22,000 to €1,000 per QALY at age 75 for the Netherlands and Greece, respectively. Keep in mind, as we assume medical costs as a proxy estimation for unrelated medical costs in our study, the magnitude of the results is overestimated to some degree. The impact of including non-medical costs is shown in the middle graph of Fig. 3. At all ages, the impact of non-medical costs is highest for Germany and lowest for Greece. The combined impact of future unrelated medical and non-medical costs on the ICER is presented in the bottom graph of Fig. 3.

The future costs by age in Fig. 3 were also included in a CEA model by van der Pol et al. 2021 [37]. In the Dutch setting for heart failure patients at age of 65, the undiscounted deterministic baseline result is €19,200 per QALY (rounded to the nearest hundredth). Including both unrelated medical and non-medical costs would result in the ICERs increasing to about €43,000 per QALY. According to the model, the Sacubitril/valsartan treatment would result in 0.45 LYG and 0.33 QALYs. The size of the impact of Greek costs on the ICER is predictably the lowest, at about €11,600 when considering the combined costs. It is generally highest in the Netherlands and Germany, at €23,900 and €29,800 when considering the combined costs, respectively. The combined costs (unrelated medical and non-medical costs) for the UK and Spain were €20,300 and €20,100, respectively. The results showed the additional impact in the case study were of similar magnitudes to those presented in Table 2. More details on the results of the case study can be seen in the supplementary materials.

## Discussion and conclusion

The purpose of cost-effectiveness analysis is to provide decision-makers with information on the costs and benefits of the adoption of new health technologies. Often overlooked are costs related to increased survival, and a possible reason for their exclusion is likely the fact that publicly available estimates are scarce. This paper provides standardized estimates of unrelated medical and non-medical costs according to a framework for cost-effectiveness analysis first presented by Meltzer (1997) [4]. Medical costs are provided for Germany, Spain, and Greece, and non-medical costs for the UK, German, Spain, and Greece. The results show variation in the magnitude of both unrelated medical and non-medical costs between countries, as well as variation in age and sex patterns. These findings are in line with other studies in literature [9–11, 13, 14, 16–20] The impact of including future costs in the ICER varied between countries, ranging from below €1,000 and up towards €35,000 per QALY gained depending on age and which future costs are included. Looking at the decomposed cost categories, it is the unrelated medical costs that has the largest variation between countries.

The case study analyses of the Sacubitril/Valsartan treatment performed in this study indicate the economic significance of including future costs on the ICER, as the magnitudes are relatively large. Bear in mind, the impact on ICER estimates, or ‘Partial ICERs’, exclude intervention costs, productivity costs, and other cost components. We can compare the magnitude of the ‘Partial ICERs’ to generally accepted WTP thresholds to assess the impact upon cost-effectiveness. The UK and Dutch impact of both future costs (€20,300 and €23,900, respectively) form a large part or may even exceed generally accepted WTP thresholds as suggested by the individual countries’ HTA agencies (€20,000–30,000 and €20,000–80,000, respectively) [56]. Meanwhile, the Spanish impact of both future costs (€20,100) would make up 2/3 of the generally accepted WTP threshold of €30,000 [57]. This shows that despite the variation in future costs between countries, the empirical estimates still illustrate the relatively large impact of including future costs on the ICER. The inclusion may drive the ICER above WTP threshold when all other costs are accounted for and is therefore likely to affect healthcare resource decisions.

While the impact in each country is relatively large in relation to WTP thresholds, the variation in magnitude between countries is considerable. In particular, the large variation in cost components in the medical costs data input is of concern for the cross-country comparisons. Differences in health care financing may partly explain the variation in unrelated medical costs, especially between Greece and the other countries. Public financing of healthcare is a key cost input in the unrelated medical costs in this study. In addition, it affects the health and life expectancy of populations, influencing population-level estimates of quality of life and mortality [58–60]. Cyles et al*.* 2015 show Dutch and German healthcare spending (average health expenditure per capita) as roughly double the spending in Greece, similar to our estimates [61]. However, our unrelated medical costs are limited to the available public healthcare spending data, consequently omitting private healthcare spending. Hence, as the share of healthcare financing between public and private (OOP) spending varies across the countries in this article, it follows that those countries with a larger share of private healthcare financing are likely to have relatively low unrelated medical costs [61]. To this point, the literature on public healthcare spending in Greece points to an increasingly higher share of private funding and informal care giving due to austerity policies since its financial crisis [62–64]; private spending on healthcare, mainly OOP spending, approached 41% in 2015 [62]. Similarly, reports on the Spanish healthcare system show a relatively large portion of OOP spending in Spain, around 24% (EU average is around 17%) [65]. This burden of healthcare costs on the patient should theoretically be present within the data on household expenditure, however, in this study, private healthcare spending was generally excluded from the estimates to avoid double counting. The share of household spending on healthcare did not vary greatly between the countries of interest, leading us to suspect that the OOP spending in Greece and Spain is unaccounted for even here; therefore, we consider that unrelated medical costs of Greece and potentially Spain may be underestimated.

Contrary to the increasing impact of unrelated medical costs on ICERs by age, non-medical costs have relatively stable cross-country variation over the ages, with a variation of roughly €10,000. The variation in costs between may partially be explained by variation in GDP between countries, for example the GDP of the Netherlands and the UK, €53,000 and €50,000 PPP respectively, are roughly double the GDP of Greece, at €28,000 PPP. Likewise, the Dutch and British combined impact of unrelated medical and non-medical costs on ICER results are about twice the Greek result, at low ages. At higher ages, the difference in impact between these countries rises to about a threefold difference [66]. In terms of the non-medical costs, in addition to GDP differences, variation between countries can be explained by factors such as income and price level differences. For example, the UK, The Netherlands, and Germany have price levels around 20–24% above the EU average. However, Greece has a price level of 20% below the EU average [68], which is in line with the non-medical costs result for Greece in this paper.

One element we sought to investigate was the relationship of the costs with age and sex in different countries. Both unrelated medical and non-medical costs show age and sex patterns which are, in general, in line with other studies on future costs such as van Baal, Perry-Duxbury, and Kellerborg and colleagues [9–11], with some exceptions. There are between-country differences: the increase in unrelated medical costs with age is smaller in Greece and Spain compared to the other countries, in addition to being substantially lower in magnitude. In both these countries the unrelated medical costs merely double over age, while the other countries’ unrelated medical costs are more than threefold at age 75 compared to age 25. The variation may be due to the cost components of the input costs. While some estimates include cost components like long-term care and outpatient care, medical costs for Greece, and to a lesser degree Spain, capture fewer cost components than the others (see Table 1). These additional cost components may be correlated with larger consumption among the elderly, thus causing a larger increase of unrelated medical costs with age in these countries. A recent study by Kalseth et al*.* 2020 investigated the utilization of healthcare services decomposed into healthcare^8^ and long-term care^9^ across a lifespan, showing healthcare costs such as primary care (physicians), prescription medicine, and specialist somatic healthcare peaking around age 80, before decreasing sharply. As most unrelated medical cost inputs provide an average for old age, it seems likely that the averages of 75+ (Greece), 80+ (Spain), and 85+ (Germany) would capture this reduction at very old age. Long-term care on the other hand has a sharp and very large increase from age 70, supporting the notion of a larger gradient by age in unrelated medical costs results which include long-term care cost components [68]. Likewise, the lower gradient may be affected by the prevalence of OOP and informal care in Spain and Greece if this is predominantly elderly healthcare consumption [62, 65, 69]. The results for Greece also diverged somewhat as unrelated medical costs for females appeared to be lower than those of males, while this relationship is the opposite in the other countries. High consumption of informal healthcare by Greek females in old age could perhaps contribute to the explanation of this reversal of the relationship seen in our results; however, it may just as well stem from other factors such as the fact that the Greek results are only based on hospitalization expenditures [62, 69].

Regarding the interpretation of the data, an important limitation is the direct comparison of the results between countries. Methodological differences stemming from the scarcity of data, such as limited cost components in unrelated medical costs and unavailability of individual-level data for non-medical costs in some countries, have factored into the results. Furthermore, while age- and sex-consumption patterns were captured in the input data for most of the countries, the Greek age and sex pattern was approximated based on self-reported hospital admission proportions by age and sex. We were able to adjust the Spanish costs to the cost level of SHA system but were unable to make any adjustments to the Dutch (RIVM-based estimates) nor UK (HES-based estimates). This means the Dutch estimates is likely somewhat higher compared to the SHA based estimates, as RIVM includes cost components like international care, while British estimates are likely lower [10, 12]. Due to scarcity of data on the household’s composition (number of adults and children per household), we could not apply the within households’ economies of scales for all members of households for all countries, which may lead to an overestimation of non-medical costs in Greece, Germany, and Spain. Some studies find age-period-cohort effects as well as use more advanced methods such as generalized additive models and B-splines on age for non-medical costs, however, due to lack of (individual-level) data we did not consider those effects. Further, the shapes of the non-medical costs graph are in line with the PAID 3.0 tool for the Netherlands, where the results of non-medical costs are reported per capita and by age [9, 10]. Lastly, we did not include uncertainty around our costs in this report, which could be of particular interest for further research with regard to survival and cost [10].

In this paper, we provided estimates of future unrelated medical and non-medical costs for the UK, Germany, Spain, and Greece based on the methodology of Van Baal and colleagues. Hence giving researchers and health economists access to standardized estimates which can be applied in cost-effectiveness analyses in different country settings, following the recommendation of Meltzer [4]. The data presented here allow the inclusion of different components of future costs in CEA for the countries included in this study, in the same manner as performed in the case study analyses of the Sacubitril/Valsartan treatment. For other countries, the estimates presented here can serve as a proxy. Further research is required to obtain more comprehensive estimates of future costs in countries with scarcity of data. As it stands, availability and detail of cost component data being so varied, we likely underestimate the true burden of healthcare in society in some of our estimates, especially in terms of private spending on healthcare. Nonetheless, as the findings in this paper shows, the inclusion of future costs in cost-effectiveness analyses represents such a large impact that it should not be neglected.

## Supplementary Information

Below is the link to the electronic supplementary material.

Supplementary file1 (XLSX 11 KB)

## Footnotes

1 Note that in some cases the impact of including future healthcare costs may be negative. For instance, when life is extended at a very young age, the impact of inclusion may be negative, as dying at a very young age is typically accompanied with relatively high medical spending.

2 Unrelated medical costs in the Netherlands produced by Van Baal and colleagues and updated by Kellerborg and colleagues [9, 10].

3 Non- medical costs in the Netherlands produced by Kellerborg and colleagues [15].

4 Unrelated medical costs in the United Kingdom produced by Perry-Duxbury and colleagues [11].

5 Note that this and the following equations are based on CEA from a societal perspective. When a healthcare perspective applies, RMC and UMC remain important, although PC and NMC are excluded.

6 Greek EQ-5D QALE estimate scores were not available in the paper by Heijink et al*.* 2011 [34], hence Spanish EQ-5D estimates were used as a proxy estimate.

7 In the case of Greece, MC costs were derived using total aggregate hospital expenditure distributed by population and hospital admission frequency data, which were distinguished by age and sex.

8 Healthcare: primary care (physicians), prescription medicine, specialist somatic healthcare, and mental healthcare (comprising specialist psychiatric and substance abuse treatment) [68].

9 Long-term care: long-term residential (nursing home) care, short-term stays in nursing homes (including short stays for examination/treatment, (re) habilitation, other short stays, day-time and night-time stays), home nursing, home help (including practical aid, and training of daily activities, user-controlled personal assistance, meals on wheels and safety alarm), care salary, respite care, activity services (including support persons and day activities) [68].
